# How to Deal With Vaccine Breakthrough Infection With SARS-CoV-2 Variants

**DOI:** 10.3389/fpubh.2022.842303

**Published:** 2022-03-15

**Authors:** Ying Guo, Jun Meng, Caide Liu, Guosheng Chen, Yuhua Chi, Shiliang Zheng, Haixia Wang

**Affiliations:** ^1^Department of Endocrinology, Affiliated Hospital of Weifang Medical University, Weifang, China; ^2^Department of Respiratory Medicine, Affiliated Hospital of Weifang Medical University, Weifang, China; ^3^Department of General Practice, Affiliated Hospital of Weifang Medical University, Weifang, China; ^4^General Practice Teaching and Research Section, Weifang Medical University, Weifang, China; ^5^Department of Blood Transfusion, Affiliated Hospital of Weifang Medical University, Weifang, China

**Keywords:** SARS-CoV-2, variants, breakthrough, infection, Delta, virulent strains

## Abstract

Novel Coronary Pneumonia is the most infectious disease with the highest number of morbidity and mortality in 100 years. Despite aggressive and effective COVID-19 prevention and control measures, countries have been unable to stop its outbreaks. With the widespread use of vaccines, the occurrence of COVID-19 has declined markedly. April 21, 2021, New York scholars reported Vaccine Breakthrough Infections with SARS-CoV-2 Variants, which immediately attracted widespread attention. In this mini-review, we focus on the characteristics of SARS-CoV-2 and its mutant strains and vaccine breakthrough infections. We have found that outbreaks of vaccine-breaking SARS-CoV-2 Delta infections in many countries are primarily the result of declining vaccine-generated antibody titers and relaxed outbreak management measures. For this reason, we believe that the main response to vaccine-breaking infections with the SARS-CoV-2 variant is to implement a rigorous outbreak defense policy and vaccine application. Only by intensifying the current vaccination intensity, gradually improving the vaccine and its application methods, and strengthening non-pharmaceutical measures such as travel restrictions, social distancing, masking and hand hygiene, can the COVID-19 outbreak be fully controlled at an early date.

## Introduction

Severe acute respiratory syndrome coronavirus type 2 (SARS-CoV-2) is a highly contagious virus that is spread primarily by droplets or close contact and is spreading faster, in bizarre ways, and in larger numbers than anyone in the world expected (1–3). By September 2021, more than 2.2 billion cases of COVID-19 infection had been diagnosed and more than 4.6 million deaths had occurred. the COVID-19 pandemic not only poses a serious threat to human life and health, but has also significantly stalled global economic development and caused enormous losses to people around the world.

Despite aggressive and effective measures to control COVID-19, the epidemic has not yet subsided. Hopes were then raised that a vaccine could beat COVID-19 (4–6). Encouragingly, with the widespread use of vaccines in many developed countries, the incidence of COVID-19 has declined significantly (7). To date, vaccination in some countries, such as the USA and the UK, has approached the level of herd immunity. However, a new wave of COVID-19 outbreaks has recently occurred in the UK and USA, and these outbreaks are mainly due to SARS-CoV-2 Delta variant (8–12).

The critical issue of vaccine breakthroughs for Delta infections leading to new outbreaks has attracted the attention of many governments and academics. We searched the following databases: PubMed, Web of Science, EMBASE and CNKI for the search terms “SARS-CoV-2,” “COVID-19,” “Variants,” “Breakthrough,” “Delta,” “Delta,” “Infections.” A comprehensive analysis of SARS-CoV-2 mutations and vaccine breakthrough infections is presented, and active and effective countermeasures are proposed to avoid new outbreaks of the new COVID-19 epidemic.

## SARS-CoV-2 and Its Variant Strains

SARS-CoV-2 is a beta genus coronavirus with an envelope membrane. It has five basic genes, four structural proteins (N, E, M and S) and an RNA-dependent RNA polymerase (RdRp). SARS-COV-2 enters the host cell through the spinosin (S protein) on the surface of its envelope and binds to the host cell surface-specific viral receptor, angiotensin-converting enzyme 2 (ACE2), so the airway mucosa, oral mucosa, which are in communication with the outside world, and the eyelids and conjunctiva are susceptible to exposure to infection (13, 14). Because the genetic material of SARS-COV-2 is single-stranded RNA, it is characterized by rapid mutation, high genetic diversity and high prevalence. The entry of SARS-CoV-2 into host cells is mediated primarily through the binding of its receptor-binding domain (RBD) to the cellular receptor human angiotensin-converting enzyme 2 (ACE2), making the S protein a key target for antibody therapies to prevent SARS-CoV-2 virus infection and limit its spread (15). Indeed, the RBD is recognized by potentially neutralizing and diverse monoclonal antibodies, thereby providing protection by preventing SARS-CoV-2 from entering host cells and binding to ACE2 (16). The SARS-CoV-2 spike protein contains several different antigenic sites, including multiple receptor-binding domain (RBD) epitopes and non-RBD epitopes, which may also generate immune escape (17).

The survival time of SARS-COV-2 depends on its own characteristics, physical and chemical properties of the surface and the environmental conditions, such as climate, light, temperature, humidity and so on (18–20). It was reported that SARS-COV-2 survived for 7 days on the plastic surface, 4 days on the stainless steel surface at room temperature, and relatively short on the commonly used paper documents, banknotes and mail wrapping paper (20).

Viruses are prone to mutation, and the longer they are transmitted, the more mutant strains are produced. Of course, virus mutations are random and may produce milder, less pathogenic variants, but also more pathogenic and infectious variants. A study published on 24 May 2021 concluded that SARS-CoV-2 mutates almost weekly, at a rate more than 50% higher than previously thought (21). Since the COVID-19 pandemic has been ongoing for nearly two years, the multiple mutations generated by SARS-CoV-2 are natural. Mutation itself is not a bad thing, and there is no indication that mutations in SARS-CoV-2 are faster or slower than expected. So far, we cannot say whether SARS-CoV-2 is becoming increasingly lethal and infectious.

The World Health Organization (WHO) classifies the various variants of the SARS-CoV-2 virus according to disease severity, infectivity, public health implications such as city closures, masks, social distancing and timely diagnosis, early treatment and vaccine application. On 31 May 2021, the WHO announced that it would use the Greek alphabet to identify important new coronavirus mutants and published a list of current mutants of concern (VOC) and mutants of interest (VOI) designations (22). Variants of Concern (VOC) are those that have been shown to cause more severe disease, to be more infectious, or to have an impact on the response. Four are currently classified as “Variants of Concern”: Alpha (B.1.1.7), first discovered in the UK, Beta (B.1.351) in South Africa, Gamma (P.1) in Brazil and Delta (B.1.617.1) in India (22).

The Delta variant has been described by WHO as the fastest spreading and most adaptive” virus due to its outstanding infectivity and immune escape profile. It is highly transmissible, has a high viral load and is highly pathogenic. It has a relatively short incubation period in the body, with onset of disease in two to three days, and in some cases in 24 h. The virus is highly infectious, and early in transmission, SARS-CoV-2 Delta VOC was transmitted for four generations in only 10 days, with a marked increase in viral load characteristic of the virus (23). Patients infected with the Delta strain were first detected on average four days after exposure to the virus, i.e. an incubation period of four days, compared to six days for patients infected with the original strain, suggesting a much faster replication rate of the Delta strain (23). Moreover, the virulence of the Delta strain is significantly enhanced, with patients infected with the Delta strain having a viral load 1,260 times higher than that of patients with the common COVID-19 strain and a turnaround time of 13 to 15 days, significantly longer than the 7 to 9 days of the original strain (23). Many studies have confirmed that the Delta strain is significantly more virulent and infectious and is the main SARS-CoV-2 variant responsible for this year's world pandemic (24, 25). Recently, a new variant, named as Omicron, was detected in South Africa in the November 2021.

## Vaccine Breakthrough for SARS-CoV-2 Delta Infection

An article published online 21 April by Hacisuleyman et al. (26) reinforces the understanding of SARS-CoV-2 mutation and vaccine breakthrough. Two women aged over 50 years were identified among 417 people who had received a second dose of BNT162b2 (Pfizer Biotech) or mRNA-1273 (Modena) vaccine at least 2 weeks earlier Presence of vaccine breakthrough infection. This suggests that despite high levels of neutralizing antibodies (where the vaccine remains effective), infection by variant viruses can occur even at high viral loads. Therefore there is still a potential risk of infection after vaccination and precautions for COVID-19 infection are also taken (26). Around a wedding outside Houston, Texas, two patients from India may have transmitted the delta-mutant virus to other guests, six of whom were fully vaccinated (Pfizer BNT162b2, Moderna mRNA-1273 and Covaxin BBV152). This means that the delta variant is highly transmissible and prone to vaccine breakthrough infections (25).

In the early days of COVID-19 some scholars proposed the use of herd immunization strategies to combat the epidemic. As COVID-19 outbreaks continued to cause high morbidity and mortality in many countries, herd immunization was found to be too costly to be feasible (25, 27). With the introduction of the vaccine, the incidence of COVID-19 began to decline markedly in many countries, and in late March 2021 India liberalized some public events, only to see a major outbreak of SARS-CoV-2 Delta infection began in India as local temperatures rose (28). The use of the vaccine caused many countries to let down their guard to eliminate non-pharmaceutical precautions such as travel restrictions, and the population grew tired of the COVID-19 outbreak and loosened their outbreak control measures. Because of the spillover of SARS-CoV-2 Delta infection in India and the simultaneous removal of travel restrictions for the outbreak in many countries such as Israel, the United Kingdom and the United States, there was a rapid outbreak of SARS-CoV-2 Delta infection in these countries (8–10, 29).

These variants show reduced neutralization *in vitro* by antibodies raised in humans in response to SARS-CoV-2 infection or vaccination (30). Indeed, early studies have reported that antibodies persist for about six months after SARS-CoV-2 infection in humans (31). These observations highlight the need to better understand the breadth of antibody responses induced by SARS-CoV-2 vaccine and to potentially adapt prophylactic and therapeutic agents to respond to emerging variants (32). Although vaccination is widespread in many countries, by the second half of 2021 it will have been partially half a decade since antibodies produced by the vaccine have declined significantly and vaccine breakthrough SARS-CoV-2 Delta infections have occurred successively (8–10, 23, 25, 33–42). See Table 1.

**Table 1:** Reports of vaccine breakthrough infection.

**Time**	**Location**	**Area**	**Country**	**Staff**	**Vaccination status**	**Incidence rate**	**Characteristics and prognosis**	**References**
Dec, 2020–Aug, 2021	NA	Eight U.S. Locations	US	Frontline Workers	83% vaccinated	1.0%^*^	89.7% symptomatic	Fowlkes et al. (34)
Mar–Jun, 2021	NA	17 states	India	COVID-19 cases	2 doses 87.4%; 1 dose 12.6%	86.7%	0.4% fatality	Gupta et al. (35)
Apr, 2021	care home	London	UK	Resident staff	2 dose 75.6%; 1 dose 4.9%	63.6%^*^	No severe	Williams et al. (8)
Apr, 2021	a wedding	Houston, Texas	US	family members	fully vaccinated	<6.0%^*^	severe <1%, died <1%	Farinholt et al. (25)
Apr 01–Jun 06, 2021	NA	Scotland	UK	COVID-19 cases	2 doses 39.4%; 1 dose 59.4%	40.2%	hospital admissions 1.9%	Sheikh et al. (36)
Apr 15–May 27, 2021	Gymnastics Facility	Oklahoma	US	Gymnast, staff, households	38% fully vaccinated	45.0%	NA	Dougherty et al. (37)
May 01–Jul 25, 2021	NA	Los Angeles	US	COVID-19 cases	25.3% fully vaccinated,3.3% Partial vaccinated	91.2%	More infected and hospitalized	Griffin et al. (38)
May 03–May 07, 2021	NA	Chennai	India	COVID-19 cases	2 doses 21.0%,1 dose 44.7%	70.1%	Moderate/Severe 15.0%,Died 0.8%	Thangaraj et al. (39)
May 21–Jun 18, 2021	NA	Guangzhou	China	Delta VOC infected	6.3% fully vaccinated	100%	5.0% were severe, and 7.5% were critical	Wang et al. (23)
May 24–Jun 01, 2021	Elementary School	Marin County, California	US	Student, teacher, parents	10.7% fully vaccinated	12.0%^*^	No persons were hospitalized	Lam-Hine et al. (9)
Jun 14–Jul 30, 2021	community	three regions	France	COVID-19 cases	11.2% vaccinated	90.8%	NA	Blanquart et al. (40)
Jul–Aug, 2021	Prison	Texas	US	Incarcerated Person	79.4% fully vaccinated	69.7%^*^	One (3%) died (unvaccinated)	Hagan et al. (10)
Jul, 2021	Nosocomial	Meir Medical Center	Israel	Patient, staff and families	96.2% vaccinated	15.7%^*^	19.0% severe, 14.3% critical and 11.9% died	Shitrit et al. (41)
NA	family	NA	France	family cluster	25.0% vaccinated	75%^*^	Uninfected:the vaccinated	Nathan et al. (42)
NA	hospital	NA	India	health career	2 doses:29.8%;1 dose:58.5%	1.6%^*^	NA	Rana et al. (43)

* *Refers to the breakthrough infection rate*.

SARS-CoV-2 vaccine protection is unlikely to be 100% (44, 45), and it makes sense that vaccine breakthrough SARS-CoV-2 Delta infections would occur as SARS-CoV-2 Delta variants reduce the rate of vaccine protection. Although SARS-CoV-2 Delta variants have caused multiple outbreaks of vaccine breakthrough infections, vaccine protection against SARS-CoV-2 Delta variants remains effective, particularly in preventing severe illness and reducing mortality. After two doses of BNT162b2 mRNA vaccine 2, 94% efficacy against symptomatic novel coronavirus was achieved. Vaccine efficacy estimates for the study results from day 14 to day 20 after the first dose and day 7 or more after the second dose were as follows: 46% and 92% for documented infections; 57 and 94% for symptomatic neocoronavirus-19; 74 and 87% for hospitalization; and 62 and 92% for severe disease, respectively. The estimated effectiveness of prevention of neocoronavirus-19 mortality was 72% from day 14 to day 20 after the first dose. Estimates of effectiveness for specific subgroups assessed for documented infections and symptomatic neocoronavirus-19 were consistent across age groups, with slightly lower potential effectiveness in those with multiple coexisting diseases (35). One dose of the vaccine (BNT162b2 or ChAdOx1 nCoV-19) was significantly less effective against the delta variant at 30.7% than against the alpha variant at 48.7%; the results were similar for both vaccines. Two doses of BNT162b2 vaccine provided 93.7% protection against the alpha variant and 88.0% protection against the delta variant. Two doses of the ChAdOx1 nCoV-19 vaccine provided 74.5% protection against the alpha variant and 67.0% protection against the delta variant (43). The vaccine and the multiple monoclonal antibodies against SARS coronavirus produced after infection acted together with other antibodies to produce beneficial effects (46).

Israel, which has the highest vaccination rate, had an outbreak of SARS-CoV-2 Delta infection in 2021.7 (29). The fact that the outbreak of SARS-CoV-2 Delta infection was quickly controlled in Israel following an enhanced control strategy and a third dose of vaccination also demonstrates that the vaccine remains effective against SARS-CoV-2 Delta infection. In the face of the relative weakness of the delta strain in the face of the vaccine, we humans would face a major crisis if a mutant virus emerged that had both the transmissibility of the delta strain and the tolerance of the beta strain.

## Proactive Response to Vaccine Breakthrough of SARS-CoV-2 Delta Infection

It is an indisputable fact that vaccine breakthrough of SARS-CoV-2 Delta infection has caused a new outbreak. In the face of this problem, we need to take a variety of positive measures to actively respond in order to control the outbreak caused by SARS-CoV-2 Delta infection.

Neutralizing antibodies and cellular immunity against SARS-CoV-2 produced after COVID-19 infection and after vaccination are also important. Some studies have reported that the immune response generated by simple colds caused by coronaviruses also has a role against SARS-CoV-2, although it is limited (47). Neutralizing antibody against SARS-CoV-2 produced after vaccination usually show a gradual decline from three months onwards, with a marked decrease after six months (31). Neutralizing antibodies against SARS-CoV-2 have almost no effect six months after vaccination, but cellular immunity remains (48). The cellular immune response is primarily a T-cell response, including cytotoxic T cells and helper T cells. Memory B cells can survive for a long time after infection with COVID-19 or vaccination, especially after an MRNA vaccination. It can be reactivated and produce antibodies when the pathogen reinvades, even if the level of antibodies in the serum is low (48).

Although experts in countries such as Israel and the United States disagree on whether to give a third dose of vaccine, most scholars advocate the application of booster vaccinations. The more the vaccine is used over time, the more research findings show that the protective power of the vaccine decreases over time, making it necessary to give boosters after more than six months of vaccination in special populations (49). Therefore, until the new SARS-CoV-2 outbreak is under control, when there is an outbreak, we still recommend a third dose of vaccine six months after vaccination. Frontline staff in customs, airports, hospitals and CDC are to receive their third dose of vaccine. Hospital staff in fever clinics, infection units, respiratory units, emergency departments and nucleic acid testing units are given the third dose of the vaccine. The epidemic will not go away for a short time, maybe it is inevitable to accept the third vaccine injection for the adult. There were no significant side effects in vaccinated American children (50). We encourage that children were also advised to be vaccinated, When implementing a third vaccination strategy, it is best to administer the third dose using a vaccine with a different route of action. The vaccines used vary from country to country, with the most used being the Messenger RNA (MRNA) vaccine: BNT162b2 (Pfizer-BioNTech) mRNA vaccine, mRNA-1273 (Moderna) mRNA vaccine; Adenovirus vaccine: AZD1222;Inactivated vaccine: BIBP Corona VAC (Sinopharm), Corona VAC (SLC), Sputnik V. Studies have reported higher antibody production from different types of vaccines or from a combination of vaccines produced by different companies, suggesting that the actual effect on protection against COVID-19 infection may be better. This suggests a possible better practical effect against COVID-19 infection (51).

COVID-19 outbreaks can spread between different countries. Considering that underdeveloped countries such as Africa have difficulty accessing the vaccine for economic reasons, some experts have called for a vaccine equity programme to be implemented for people in poor countries who have not been vaccinated (52). We believe that equitable distribution of vaccines is necessary and that it shows fairness and love for humanity, but vaccines should be given priority to areas with severe outbreaks. At present, the flow of social personnel is fast, and the hardest hit area of the epidemic is the major source of infection. Giving priority to the use of vaccines in the hardest hit areas is a sign of being responsible for the global epidemic. Only in this way will we receive better results.

The development of an effective COVID-19 vaccine is a global imperative. Rapid immunization of the entire human population against widespread, evolving highly pathogenic viruses is an unprecedented challenge, and different vaccine approaches are being sought. As viruses mutate, it is even more important for scholars to work on improving vaccine aspects by targeting vaccine changes to mutated viral genes that are highly infectious and spread quickly enough to cause outbreaks or cause severe disease, or by pre-programming vaccines to target which gene segments are prone to mutations such as P384L, K417N, E484K, N501Y (53).

Current technology is capable of rapidly setting up a genetic vaccine based on the SARS-CoV-2 variant, but phase III clinical trials will take some time. Even so, vaccine experts will have to do their best to meet practical needs in the future (54, 55).

A study in September 2020 showed that nebulized Ad5-nCoV was well-tolerated, with two doses of nebulized Ad5-nCoV inducing a neutralizing antibody response similar to one dose of intramuscular injection. Nebulized booster vaccination induced a strong IgG and neutralizing antibody response 28 days after the first intramuscular injection (56). Prophylactic intranasal application of DNA vaccine significantly reduces pulmonary infection with SARS-CoV-2 via the nose. Because SARS-CoV-2 is transmitted by droplet and close contact transmission and the nose is the primary gateway to the respiratory system, prophylactic intranasal (vaccine, etc.) administration increases local defenses and resistance to disease in the respiratory tract and may produce good results when applied in conjunction with vaccination (57). Although this vaccine is restricted to ages 5–49, the majority of COVID-19 patients are in this age group. The injection-free nebulized inhalation vaccine is readily accepted by the general public and is necessary as a complementary means to injectable vaccines. In high-risk groups, especially (58) those with occupational exposure in medical personnel, subcutaneous injection of the combination of monoclonal antibodies casirivimab and imdevimab REGEN-COV is effective in preventing SARS-CoV-2 infection.

The traditional approach to infectious disease control focuses on controlling the source of infection and cutting off the route of transmission. This approach is better applied in countries such as China and New Zealand, so that the COVID-19 outbreak in these countries is well-controlled. In August 2021, there was an incident of SARS-CoV-2 Delta infection in Nanjing, China, transmitted through an airfield. Housekeeping staff at the airport were in a high-risk group for SARS-CoV-2 infection and they were routinely given two doses of vaccine. The housekeeping staff at Nanjing Airport were SARS-CoV-2 Delta breakthrough vaccine infected. They acquired the infection while cleaning the aircraft flown by SARS-CoV-2 Delta infected persons. SARS-CoV-2 Delta has since spread rapidly through airports to cities such as Yangzhou, Zhangjiajie, Chengdu and Dalian, resulting in over a thousand SARS-CoV-2 Delta infections across the country (59). It was with non-pharmaceutical interventions that China was able to completely control the spread of SARS-CoV-2 Delta infection in less than two months.

Non-pharmacological interventions to prevent COVID-19 are a low-tech approach, but it is difficult to enter the modern world with concrete implementation. This is because times have evolved, transport is easy and interactions between countries and people are intensive, conditions that can accelerate the spread of COVID-19 epidemics (60, 61). Some populations are not aware of travel restrictions and some of the measures to prevent and control the epidemic, so it is difficult to do so. We believe that scientists should step up their calls for governments to develop scientifically rigorous epidemic preparedness policies and to use a variety of forms to guide all people to take sensible and correct precautions. Although restricting people's travel can cause a lot of inconvenience, it can lead to long-term freedom, convenience and health. Only different regions and countries should share and learn from the experience of fighting the epidemic and work together to tackle the SARS-CoV-2 breakthrough vaccine infection.

## Conclusion

For vaccine breakthrough infection, we have taken the following measures: Firstly, we must spread the use of the vaccine as soon as possible and further optimize and strengthen the quality of the vaccine; secondly, we must restrict travel in areas where the epidemic has struck, cancel or do our best to curtail gatherings such as conferences, competitions and holiday celebrations, as well as avoid public transport, minimize going out and all kinds of social activities, keep social distance, wear masks and hand hygiene outside, etc. Only in this way will it be possible to put SARS-CoV-2 in Pandora's box as soon as possible.

## Author Contributions

YG was responsible for the conception and writing of the paper. JM for the design of the paper. CL and GC for the analysis of the data and the production of the figures. YC for the selection of references for the paper. SZ for the overall ideas. All authors contributed to the article and approved the submitted version.

## Conflict of Interest

The authors declare that the research was conducted in the absence of any commercial or financial relationships that could be construed as a potential conflict of interest.

## Publisher's Note

All claims expressed in this article are solely those of the authors and do not necessarily represent those of their affiliated organizations, or those of the publisher, the editors and the reviewers. Any product that may be evaluated in this article, or claim that may be made by its manufacturer, is not guaranteed or endorsed by the publisher.

## References

[1] Wu F Zhao S Yu B Chen Y-M Wang W Song Z-G. A new coronavirus associated with human respiratory disease in China. Nature. (2020) 579:265–9. 10.1038/s41586-020-2008-3 32015508 PMC7094943

[2] Zhu N Zhang D Wang W Li X Yang B Song J. A Novel Coronavirus from Patients with Pneumonia in China, 2019. N Engl J Med. (2020) 382:727–33. 10.1056/NEJMoa2001017 31978945 PMC7092803

[3] Zhou P Yang X-L Wang X-G Hu B Zhang L Zhang W. A pneumonia outbreak associated with a new coronavirus of probable bat origin. Nature. (2020) 579:270–3. 10.1038/s41586-020-2012-7 32015507 PMC7095418

[4] Guidry JPD Laestadius LI Vraga EK Miller CA Perrin PB Burton CW. Willingness to get the COVID-19 vaccine with and without emergency use authorization. Am J Infect Control. (2021) 49:137–42. 10.1016/j.ajic.2020.11.018 33227323 PMC7677682

[5] Tumban E. Lead SARS-CoV-2 candidate vaccines: expectations from phase III trials and recommendations post-vaccine approval. Viruses. (2020) 13:54. 10.3390/v13010054 33396343 PMC7824305

[6] Arora NK Das MK. COVID-19 vaccine development and the way forward. Indian J Public Health. (2020) 64:S108–11. 10.4103/ijph.IJPH_520_20 32496238

[7] Barandalla I Alvarez C Barreiro P de Mendoza C González-Crespo R Soriano V. Impact of scaling up SARS-CoV-2 vaccination on COVID-19 hospitalizations in Spain. Int J Infect Dis. (2021) 112:81–8. 10.1016/j.ijid.2021.09.022 34536609 PMC8442297

[8] Williams SV Vusirikala A Ladhani SN Fernandez Ruiz De Olano E Iyanger N Aiano F. An outbreak caused by the SARS-CoV-2 Delta (B.1.617.2) variant in a care home after partial vaccination with a single dose of the COVID-19 vaccine Vaxzevria, London, England, April 2021. Euro Surveill. (2021) 26:2100626. 10.2807/1560-7917.ES.2021.26.27.2100626 34240699 PMC8268653

[9] Lam-Hine T McCurdy SA Santora L Duncan L Corbett-Detig R Kapusinszky B. Outbreak associated with SARS-CoV-2 B.1.617.2 (Delta) variant in an elementary school - Marin County, California, May-June 2021. MMWR Morb Mortal Wkly Rep. (2021) 70:1214–9. 10.15585/mmwr.mm7035e2 34473683 PMC8422870

[10] Hagan LM McCormick DW Lee C Sleweon S Nicolae L Dixon T. Outbreak of SARS-CoV-2 B.1.617.2 (Delta) variant infections among incarcerated persons in a Federal Prison - Texas, July-August 2021. MMWR Morb Mortal Wkly Rep. (2021) 70:1349–54. 10.15585/mmwr.mm7038e3 34555009 PMC8459894

[11] Herlihy R Bamberg W Burakoff A Alden N Severson R Bush E. Rapid increase in circulation of the SARS-CoV-2 B.1.617.2 (Delta) variant - Mesa County, Colorado, April-June 2021. MMWR Morb Mortal Wkly Rep. (2021) 70:1084–7. 10.15585/mmwr.mm7032e2 34383734 PMC8360276

[12] Alizon S Haim-Boukobza S Foulongne V Verdurme L Trombert-Paolantoni S Lecorche E. Rapid spread of the SARS-CoV-2 Delta variant in some French regions, June 2021. Euro Surveill. (2021) 26:2100573. 10.2807/1560-7917.ES.2021.26.28.2100573 34269174 PMC8284044

[13] Yang Y Zhang Y Qu Y Zhang C Liu X-W Zhao M. Key residues of the receptor binding domain in the spike protein of SARS-CoV-2 mediating the interactions with ACE2: a molecular dynamics study. Nanoscale. (2021) 13:9364–70. 10.1039/D1NR01672E 33999091

[14] Halwe S Kupke A Vanshylla K Liberta F Gruell H Zehner M. Intranasal administration of a monoclonal neutralizing antibody protects mice against SARS-CoV-2 infection. Viruses. (2021) 13:1498. 10.3390/v13081498 34452363 PMC8402634

[15] Lu M Uchil PD Li W Zheng D Terry DS Gorman J. Real-time conformational dynamics of SARS-CoV-2 spikes on virus particles. Cell Host Microbe. (2020) 28:880–91.e8. 10.1101/2020.09.10.286948 33242391 PMC7664471

[16] Barnes CO West AP Huey-Tubman KE Hoffmann MAG Sharaf NG Hoffman PR. Structures of human antibodies bound to SARS-CoV-2 spike reveal common epitopes and recurrent features of antibodies. Cell. (2020) 182:828–42.e16. 10.1101/2020.05.28.121533 32645326 PMC7311918

[17] Weisblum Y Schmidt F Zhang F DaSilva J Poston D Lorenzi JC. Escape from neutralizing antibodies by SARS-CoV-2 spike protein variants. Elife. (2020) 9:e61312. 10.7554/eLife.61312 33112236 PMC7723407

[18] Wathore R Gupta A Bherwani H Labhasetwar N. Understanding air and water borne transmission and survival of coronavirus: insights and way forward for SARS-CoV-2. Sci Total Environ. (2020) 749:141486. 10.1016/j.scitotenv.2020.141486 32827813 PMC7402210

[19] Park J-E Son W-S Ryu Y Choi SB Kwon O Ahn I. Effects of temperature, humidity, and diurnal temperature range on influenza incidence in a temperate region. Influenza Other Respir Viruses. (2020) 14:11–8. 10.1111/irv.12682 31631558 PMC6928031

[20] Ren S-Y Wang W-B Hao Y-G Zhang H-R Wang Z-C Chen Y-L. Stability and infectivity of coronaviruses in inanimate environments. World J Clin Cases. (2020) 8:1391–9. 10.12998/wjcc.v8.i8.1391 32368532 PMC7190947

[21] Morales AC Rice AM Ho AT Mordstein C Mühlhausen S Watson S. Causes and Consequences of purifying selection on SARS-CoV-2. Genome Biol Evol. (2021) 13:evab196. 10.1093/gbe/evab196 34427640 PMC8504154

[22] Konings F Perkins MD Kuhn JH Pallen MJ Alm EJ Archer BN. SARS-CoV-2 variants of interest and concern naming scheme conducive for global discourse. Nat Microbiol. (2021) 6:821–3. 10.1038/s41564-021-00932-w 34108654

[23] Wang Y Chen R Hu F Lan Y Yang Z Zhan C. Transmission, viral kinetics and clinical characteristics of the emergent SARS-CoV-2 Delta VOC in Guangzhou, China. EClinicalMedicine. (2021) 40:101129. 10.1016/j.eclinm.2021.101129 34541481 PMC8435265

[24] Baj A Novazzi F Pasciuta R Genoni A Ferrante FD Valli M. Breakthrough infections of E484K-harboring SARS-CoV-2 Delta variant, Lombardy, Italy. Emerg Infect Dis. (2021) 27:3180–2. 10.3201/eid2712.211792 34499599 PMC8632179

[25] Farinholt T Doddapaneni H Qin X Menon V Meng Q Metcalf G. Transmission event of SARS-CoV-2 delta variant reveals multiple vaccine breakthrough infections. BMC Med. (2021) 19:255. 10.1186/s12916-021-02103-4 34593004 PMC8483940

[26] Hacisuleyman E Hale C Saito Y Blachere NE Bergh M Conlon EG. Vaccine breakthrough infections with SARS-CoV-2 variants. N Engl J Med. (2021) 384:2212–8. 10.1056/NEJMoa2105000 33882219 PMC8117968

[27] Fontanet A Cauchemez S. COVID-19 herd immunity: where are we? Nat Rev Immunol. (2020) 20:583–4. 10.1038/s41577-020-00451-5 32908300 PMC7480627

[28] Brett TS Rohani P. Transmission dynamics reveal the impracticality of COVID-19 herd immunity strategies. Proc Natl Acad Sci U S A. (2020) 117:25897–903. 10.1073/pnas.2008087117 32963094 PMC7568326

[29] Golechha M Panigrahy RK. COVID-19 and heatwaves: a double whammy for Indian cities. Lancet Planet Health. (2020) 4:e315–6. 10.1016/S2542-5196(20)30170-4 32730749 PMC7384774

[30] Wadman M. Israel's grim warning: delta can overwhelm shots. Science. (2021) 373:838–9. 10.1126/science.373.6557.838 34413215

[31] Dehgani-Mobaraki P Zaidi AK Yadav N Floridi A Floridi E. Longitudinal observation of antibody responses for 14 months after SARS-CoV-2 infection. Clin Immunol. (2021) 230:108814. 10.1016/j.clim.2021.108814 34343708 PMC8325385

[32] Bichara CDA da Silva Graça Amoras E Vaz GL da Silva Torres MK Queiroz MAF. do Amaral IPC, et al. Dynamics of anti-SARS-CoV-2 IgG antibodies post-COVID-19 in a Brazilian Amazon population. BMC Infect Dis. (2021) 21:443. 10.1186/s12879-021-06156-x 33992073 PMC8122196

[33] Moradi G Mohamadi Bolbanabad A Ahmadi S Aghaei A Bahrami F Veysi A. Persistence assessment of SARS-CoV-2-specific IgG antibody in recovered COVID-19 individuals and its association with clinical symptoms and disease severity: a prospective longitudinal cohort study. Int Immunopharmacol. (2021) 98:107893. 10.1016/j.intimp.2021.107893 34174700 PMC8206627

[34] Fowlkes A Gaglani M Groover K Thiese MS Tyner H Ellingson K. Effectiveness of COVID-19 vaccines in preventing SARS-CoV-2 infection among frontline workers before and during B.1.617.2 (Delta) variant Predominance - Eight U.S. Locations, December 2020-August 2021. MMWR Morb Mortal Wkly Rep. (2021) 70:1167–9. 10.15585/mmwr.mm7034e4 34437521 PMC8389394

[35] Gupta N Kaur H Yadav PD Mukhopadhyay L Sahay RR Kumar A. Clinical characterization and genomic analysis of samples from COVID-19 breakthrough infections during the second wave among the various states of India. Viruses. (2021) 13:1782. 10.3390/v13091782 34578363 PMC8472862

[36] Sheikh A McMenamin J Taylor B Robertson C. SARS-CoV-2 Delta VOC in Scotland: demographics, risk of hospital admission, and vaccine effectiveness. Lancet. (2021) 397:2461–2. 10.1016/S0140-6736(21)01358-1 34139198 PMC8201647

[37] Dougherty K Mannell M Naqvi O Matson D Stone J. SARS-CoV-2 B.1.617.2 (Delta) Variant COVID-19 Outbreak Associated with a Gymnastics Facility - Oklahoma, April-May 2021. MMWR Morb Mortal Wkly Rep. (2021) 70:1004–7. 10.15585/mmwr.mm7028e2 34264910 PMC8314708

[38] Griffin JB Haddix M Danza P Fisher R Koo TH Traub E. SARS-CoV-2 infections and hospitalizations among persons aged ≥16 years, by vaccination status - Los Angeles County, California, May 1-July 25, 2021. MMWR Morb Mortal Wkly Rep. (2021) 70:1170–6. 10.15585/mmwr.mm7034e5 34437525 PMC8389389

[39] Thangaraj JWV Yadav P Kumar CG Shete A Nyayanit DA Rani DS. Predominance of delta variant among the COVID-19 vaccinated and unvaccinated individuals, India, May 2021. J Infect. (2021) 84:94–118. 10.1016/j.jinf.2021.08.006 34364949 PMC8343391

[40] Blanquart F Abad C Ambroise J Bernard M Cosentino G Giannoli J-M. Characterisation of vaccine breakthrough infections of SARS-CoV-2 Delta and Alpha variants and within-host viral load dynamics in the community, France, June to July 2021. Euro Surveill. (2021) 26:2100824. 10.2807/1560-7917.ES.2021.26.37.2100824 34533119 PMC8447828

[41] Shitrit P Zuckerman NS Mor O Gottesman B-S Chowers M. Nosocomial outbreak caused by the SARS-CoV-2 Delta variant in a highly vaccinated population, Israel, July 2021. Euro Surveill. (2021) 26:2100822. 10.2807/1560-7917.ES.2021.26.39.2100822 34596015 PMC8485578

[42] Nathan N Prevost B Lambert S Schnuriger A Corvol H. Severe acute respiratory syndrome coronavirus 2 variant delta infects all 6 siblings but spares comirnaty (BNT162b2, BioNTech/Pfizer)-vaccinated parents. J Infect Dis. (2021) 224:1984–6. 10.1093/infdis/jiab410 34409999 PMC8499735

[43] Rana K Mohindra R Pinnaka L. Vaccine breakthrough infections with SARS-CoV-2 variants. N Engl J Med. (2021) 385:e7. 10.1056/NEJMc2107808 34077641

[44] Lipsitch M Krammer F Regev-Yochay G Lustig Y Balicer RD. SARS-CoV-2 breakthrough infections in vaccinated individuals: measurement, causes and impact. Nat Rev Immunol. (2022) 22:57–65. 10.1038/s41577-021-00662-4 34876702 PMC8649989

[45] Bates TA McBride SK Winders B Schoen D Trautmann L Curlin ME. Antibody response and variant cross-neutralization after SARS-CoV-2 breakthrough infection. JAMA. (2022) 327:179–81. 10.1001/jama.2021.22898 34914825 PMC8678894

[46] Dagan N Barda N Kepten E Miron O Perchik S Katz MA. BNT162b2 mRNA Covid-19 vaccine in a nationwide mass vaccination setting. N Engl J Med. (2021) 384:1412–23. 10.1056/NEJMoa2101765 33626250 PMC7944975

[47] Chen RE Zhang X Case JB Winkler ES Liu Y VanBlargan LA. Resistance of SARS-CoV-2 variants to neutralization by monoclonal and serum-derived polyclonal antibodies. Nat Med. (2021) 27:717–26. 10.1038/s41591-021-01294-w 33664494 PMC8058618

[48] Loyal L Braun J Henze L Kruse B Dingeldey M Reimer U. Cross-reactive CD4 T cells enhance SARS-CoV-2 immune responses upon infection and vaccination. Science. (2021) 374:eabh1823. 10.1126/science.abh1823 34465633 PMC10026850

[49] Schmidt T Klemis V Schub D Mihm J Hielscher F Marx S. Immunogenicity and reactogenicity of heterologous ChAdOx1 nCoV-19/mRNA vaccination. Nat Med. (2021) 27:1530–5. 10.1038/s41591-021-01464-w 34312554 PMC8440177

[50] American Academy of Pediatrics. Children and COVID-19: State-Level Data Report. (2022). Available online at: https://www.aap.org/en/pages/2019-novel-coronavirus-covid-19-infections/children-and-covid-19-state-level-data-report (accessed January 28, 2022).

[51] Khan WH Hashmi Z Goel A Ahmad R Gupta K Khan N. COVID-19 pandemic and vaccines update on challenges and resolutions. Front Cell Infect Microbiol. (2021) 11:690621. 10.3389/fcimb.2021.690621 34568087 PMC8461057

[52] Liu X Shaw RH Stuart ASV Greenland M Aley PK Andrews NJ. Safety and immunogenicity of heterologous versus homologous prime-boost schedules with an adenoviral vectored and mRNA COVID-19 vaccine (Com-COV): a single-blind, randomised, non-inferiority trial. Lancet. (2021) 398:856–69. 10.1016/S0140-6736(21)01694-9 34370971 PMC8346248

[53] Al-Oraibi A Martin CA Hassan O Wickramage K Nellums LB Pareek M. Migrant health is public health: a call for equitable access to COVID-19 vaccines. Lancet Public Health. (2021) 6:e144. 10.1016/S2468-2667(21)00031-1 33640075 PMC8130913

[54] Wang R Chen J Hozumi Y Yin C Wei G-W. Emerging vaccine-breakthrough SARS-CoV-2 variants. Arxiv [preprint].arxiv:2109.04509v1. (2021). 10.1021/acsinfecdis.1c00557 35133792 PMC8848511

[55] Kustin T Harel N Finkel U Perchik S Harari S Tahor M. Evidence for increased breakthrough rates of SARS-CoV-2 variants of concern in BNT162b2-mRNA-vaccinated individuals. Nat Med. (2021) 27:1379–84. 10.1038/s41591-021-01413-7 34127854 PMC8363499

[56] Zhu F-C Guan X-H Li Y-H Huang J-Y Jiang T Hou L-H. Immunogenicity and safety of a recombinant adenovirus type-5-vectored COVID-19 vaccine in healthy adults aged 18 years or older: a randomised, double-blind, placebo-controlled, phase 2 trial. Lancet. (2020) 396:479–88. 10.1016/S0140-6736(20)31605-6 32702299 PMC7836858

[57] Zhu F-C Li Y-H Guan X-H Hou L-H Wang W-J Li J-X. Safety, tolerability, and immunogenicity of a recombinant adenovirus type-5 vectored COVID-19 vaccine: a dose-escalation, open-label, non-randomised, first-in-human trial. Lancet. (2020) 395:1845–54. 10.1016/S0140-6736(20)31208-3 32450106 PMC7255193

[58] Zhou D Chan JF-W Zhou B Zhou R Li S Shan S. Robust SARS-CoV-2 infection in nasal turbinates after treatment with systemic neutralizing antibodies. Cell Host Microbe. (2021) 29:551–63.e5. 10.1016/j.chom.2021.02.019 33657424 PMC7904446

[59] O'Brien MP Forleo-Neto E Musser BJ Isa F Chan K-C Sarkar N. Subcutaneous REGEN-COV antibody combination to prevent Covid-19. N Engl J Med. (2021) 385:1184–95. 10.1056/NEJMoa2109682 34347950 PMC8362593

[60] Zhou L Nie K Zhao H Zhao X Ye B Wang J. Eleven COVID-19 outbreaks with local transmissions caused by the imported SARS-CoV-2 Delta VOC - China, July-August, 2021. China CDC Wkly. (2021) 3:863–8. 10.46234/ccdcw2021.213 34703643 PMC8521157

[61] Song SW Kim D Park JY Lee S. Symptoms and characteristics which require attention during COVID-19 screening at a port of entry. J Korean Med Sci. (2021) 36:e14. 10.3346/jkms.2021.36.e14 33429473 PMC7801149

